# Unusual Presentation of Incarcerated True Parahiatal Hernia: Management of a Rare Clinical Entity

**DOI:** 10.7759/cureus.57152

**Published:** 2024-03-28

**Authors:** Guo Hou Loo, Guhan Muthkumaran, Nik Ritza Kosai

**Affiliations:** 1 Upper Gastrointestinal (GI) and Metabolic Surgery Unit, Department of Surgery, Universiti Kebangsaan Malaysia Medical Centre, Kuala Lumpur, MYS; 2 Upper Gastrointestinal (GI) and Metabolic Surgery Unit, Department of Surgery, Hospital Canselor Tuanku Muhriz, Universiti Kebangsaan Malaysia, Kuala Lumpur, MYS

**Keywords:** fundoplication, tension free mesh repair, upper gi, incarcerated hernia, paraoesophageal hernia, gerd, parahiatal hernia

## Abstract

True parahiatal hernia is a type of diaphragmatic hernia in which herniation occurs through a defect in the diaphragm, adjacent to the normal oesophageal hiatus. Its reported incidence is very rare, and it is commonly misdiagnosed as paraoesophageal hernia. Although the clinical distinction between paraoesophageal and parahiatal hernia is difficult, it is essential to recognise these two separate entities clinically as their management differs.

Clinical presentation of parahiatal hernia includes symptoms related to gastro-oesophageal reflux disease (GERD). Patients may also present emergently with symptoms of respiratory distress and chest symptoms. With that in mind, we describe a compelling case of a young lady who initially presented with symptoms suggestive of acute coronary syndrome. However, she was found to have an incarcerated parahiatal hernia.

## Introduction

True parahiatal hernia is a rare form of diaphragmatic hernia [1]. In contrast to paraoesophageal hernia, true parahiatal hernia occurs through a diaphragmatic defect situated adjacent to but distinct from an anatomically intact oesophageal hiatus [2]. The estimated incidence of parahiatal hernia is 0.2% in patients undergoing fundoplication. Parahiatal hernia is classified as primary (congenital) and secondary (acquired) [2]. Secondary parahiatal hernia is the more common type and usually occurs after oesophageal surgery. This is possibly due to disturbance to the normal anatomy [2].

Although the clinical distinction between paraoesophageal and parahiatal hernia is difficult, it is essential to recognize these two separate entities intraoperatively as their surgical management differs. Oftentimes, parahiatal hernia may be diagnosed as paraoesophageal hernia preoperatively as they have similar findings on imaging [3]. Clinical presentation includes symptoms related to gastro-oesophageal reflux disease (GERD), such as recurrent heartburn, epigastric pain, nausea, and retrosternal chest pain. Patients may also present emergently with symptoms of respiratory distress and chest symptoms [4]. The treatment of choice for symptomatic parahiatal hernia is surgical repair, either a laparoscopic or an open approach. The laparoscopic approach repair seems to be favoured recently, with promising outcomes [4,5].

With that in mind, we report on a 39-year-old lady who presented acutely with respiratory distress and chest symptoms. She underwent laparoscopic mesh repair for an incarcerated primary parahiatal hernia and was subsequently discharged well.

## Case presentation

A 39-year-old woman, with no co-morbidities, presented with progressive epigastric and central chest pain. She has associated upper gastrointestinal symptoms for the past three months, including reflux symptoms and early satiety. Her symptoms worsened, and she began experiencing shortness of breath during exertion and sought medical attention because of this. On presentation to the emergency department, she was tachypneic and tachycardic, but her oxygen saturation was normal. Her general systemic examination was unremarkable. 

**Figure 1:**
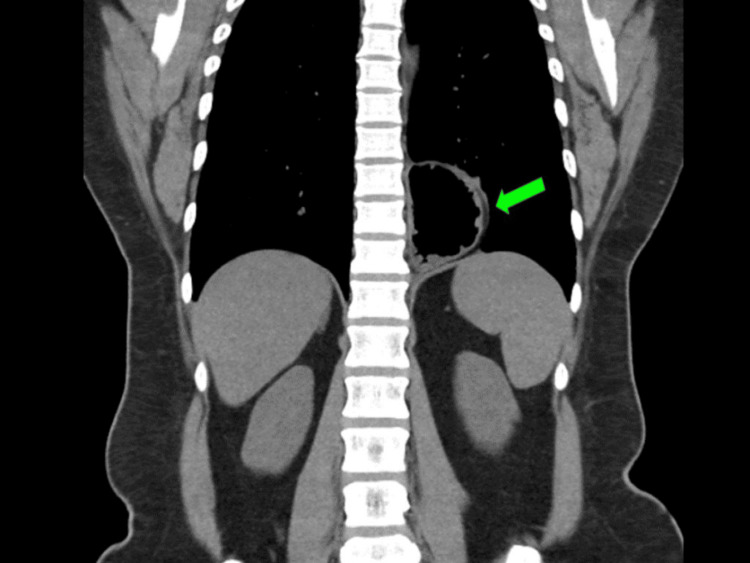
CT thorax and upper abdomen coronal view showing herniation of the stomach into the thoracic cavity (green arrow).

**Figure 2:**
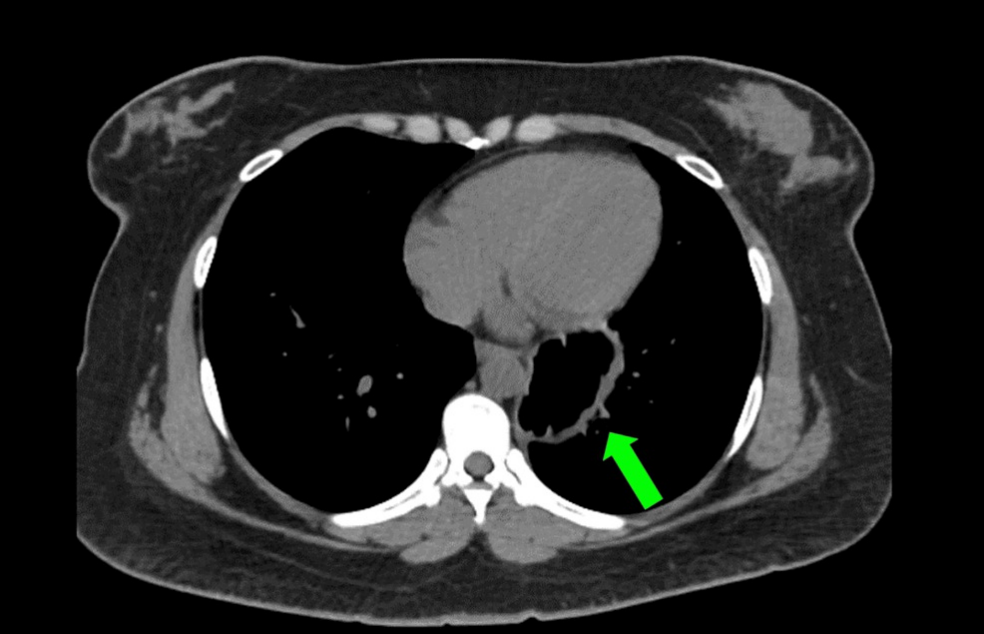
CT thorax axial view showing the herniation of body of stomach into the thoracic cavity (green arrow).

**Figure 3:**
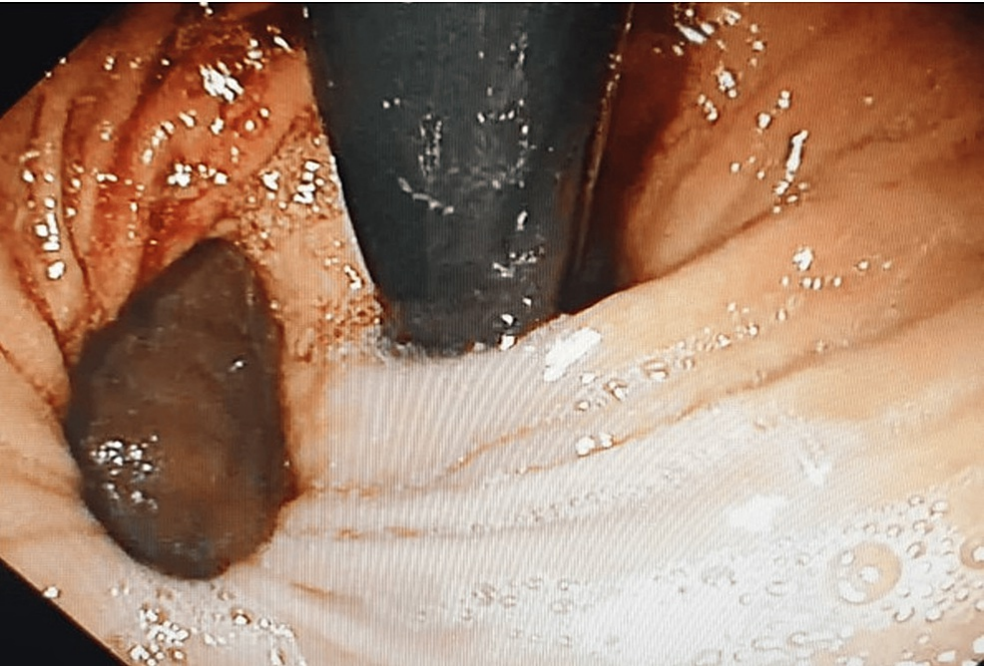
Image from upper endoscopy (on retroflexion) showing the parahiatal hernia with herniation of body of stomach.

She was initially diagnosed with acute coronary syndrome; however, her electrocardiogram and cardiac enzymes ruled that out. A chest X-ray showed a small retrocardiac gastric bubble and raised the suspicion of diaphragmatic hernia, causing her symptoms. A contrast-enhanced CT of the thorax and abdomen was performed, and it showed herniation of fundus and body of the stomach into the thoracic cavity (Figures 1, 2). An urgent oesophagogastroduodenoscopy (OGDS) was performed, and a large parahiatal hernia was seen (Figure 3). 

As she was symptomatic, she was taken to the operating theatre for the repair of the parahiatal hernia via a laparoscopic approach. A standard five-port technique for laparoscopic fundoplication procedure was employed, where the camera optical port (12 mm) was placed at the supraumbilical region, a 5-mm port at just below and left of the xiphisternum for the deployment of the Nathanson liver retractor, two 5-mm working ports at a palm’s breadth and slightly cranial on both left and right side of the supraumbilical port and finally, one 5-mm working port at the left midaxillary line two finger breadth below the left costal margin as the assistant port. Instruments used included laparoscopic bowel graspers, Maryland forceps, and an ultrasonic energy device (Harmonic™ 1100 Shears- J&J MedTech, New Brunswick, New Jersey).

**Figure 4:**
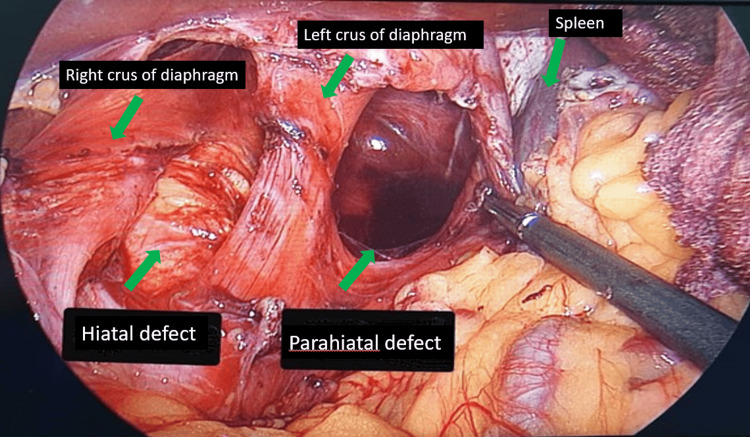
Intraoperative image showing the large parahiatal defect after reduction of hernial contents. The defect is located adjacent to the left crus of the diaphragm, and a component of hiatal defect is also seen.

**Figure 5:**
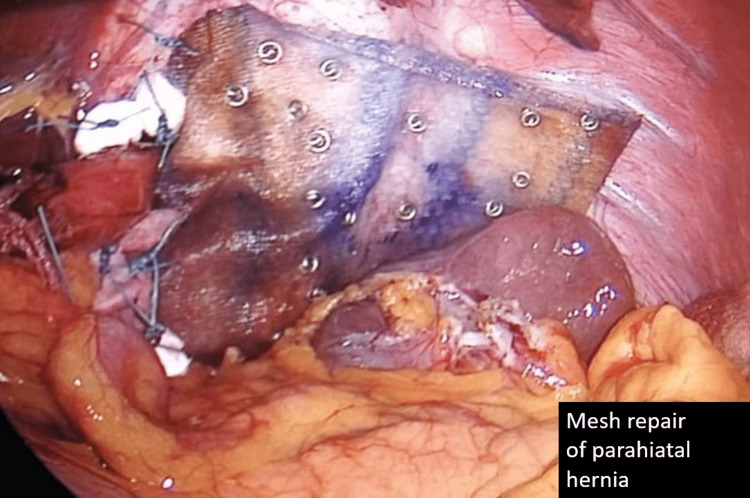
Intraoperative image of the parahiatal hernia defect after repair. The composite biocompatible mesh (with tackers) can be seen. Polyester sutures and polytetrafluoroethylene (PTFE) pledgets used to repair the hiatal hernia can be seen as well.

Intraoperatively, a 4 x 3 cm parahiatal defect was seen adjacent to the left crus of the diaphragm (Figure 4). The fundus and distal 2/3rd stomach body have herniated into the parahiatal defect. No volvulus was seen, and the stomach was viable. No other solid or hollow viscus was seen herniating into the parahiatal defect. The large parahiatal hernia defect was repaired using a composite biocompatible mesh and secured with non-absorbable polyester sutures (Figure 5). A gastropexy between greater curvature of the stomach and lateral abdominal wall was performed to prevent a recurrence. An on-table OGDS was performed to ensure normal anatomy was restored. The stomach mucosa was healthy, and the pylorus was successfully intubated up to the second part of the duodenum, confirming the restoration of normal anatomical orientation. 

Postoperatively, she was nursed in the general surgical ward and recovered uneventfully. She was allowed fluids on post-operative day one itself and progressed to a regular diet after five days. A gastrograffin study was performed 48 hours post-operatively, which showed smooth flow and no hernia recurrence. She was discharged well on postoperative day six. She was followed up six months after surgery, and she was asymptomatic upon the last review.

## Discussion

Diaphragmatic hernia and hiatal hernia are well-documented surgical entities in our literature, albeit with a low incidence, ranging from 0.17 to 6% [6]. There is another very rare subset of diaphragmatic hernia termed parahiatal hernia. Parahiatal hernia is defined as a defect in the diaphragm just lateral to either side of the right or left crus of the diaphragm [2]. Similarly, in our case, most studies also reported a left-side parahiatal hernia, suggesting it is the more common side [3]. Its exact incidence is unknown, and the majority of reported cases are from incidental findings during fundoplication. Scheidler et al. reported an incidence of 0.2% from a single Western centre, while Palanivelu et al. reported an incidence of 0.35% [2,7].

The cause of parahiatal hernia is unknown. It has been classified into primary and secondary. The primary is due to congenital weakness of the pleuroperitoneal canal caused by incomplete obliteration of the pneumoenteric recess. Secondary parahiatal hernia is commonly due to trauma but is also seen post-oesophageal surgery [8]. Obesity is a well-recognized risk factor for hiatal hernia, and it can also contribute to parahiatal hernia. Our patient's BMI was 27.3 kg/m^2, which is considered overweight but not obese. It is more common on the left side due to the protective effect of the liver on the right side [3].

Parahiatal hernia is clinically and radiologically indistinguishable from hiatal hernia [3]. As such, paraoesophageal hernia is diagnosed more frequently when a patient presents with common symptoms such as recurrent heartburn, epigastric pain, nausea, vomiting, and chest pain, coupled with a chest X-ray that may show a retrocardiac bubble. A cardiac cause of chest pain must be ruled out first, and a subsequent chest roentgenogram showing a retrocardiac gas bubble will usually prompt the clinician to further investigate for paraoesophageal hernia by performing a barium study and CT scan of the thorax. Although Scheidler attempted to describe the salient imaging features of parahiatal hernia, it is somewhat inconclusive and has never been validated by other studies [7].

It is essential to recognise the anatomical differences between paraoesophageal and parahiatal hernia repair as their management differs. The debate continues regarding whether preoperative recognition of this distinct parahiatal hernia is of any value, especially with the paradigm shifts seen with the introduction of minimally invasive surgery. The first laparoscopic repair of parahiatal hernia was reported in 1998 by Rodefeld [9]. He performed a primary repair of the defect using a non-absorbable suture. Subsequent reports have all advocated a mesh closure of the defect, owing to the principle of tension-free repair [2,3]. Notably, no complications pertaining to parahiatal hernia mesh repair have been reported in the literature. This is because the mesh placed for parahiatal hernia repair is not in direct contact with any intra-abdominal organs. Gastropexy was performed in our case to prevent hernia recurrence and to avoid close visceral contact with the mesh. Primary repair is not advocated as the weakness of the surrounding diaphragmatic muscles will likely cause the repair to be under tension, leading to a high recurrence rate [3]. Laparoscopy allows better visualisation of the peritoneal cavity and a more straightforward reduction of the hernia content. Furthermore, laparoscopy has been shown to be better than an open approach in terms of bleeding, hospital stay, post-operative pain, and improved quality of life compared to an open approach [3].

It is also essential to recognise that parahiatal hernia and sliding hiatal hernia can present simultaneously, as in our patient. Muramatsu et al. identified nine cases out of 20 where the primary parahiatal hernia was present along with a sliding hiatal hernia, which ranged from 4 cm to 5.5 cm in size [10]. Notably, either Nissen or partial wrap (anterior/posterior) fundoplication was performed for all cases with sliding hiatal hernia [10].

Parahiatal hernia is best repaired by placing a mesh due to its wide neck. In addition to mesh repair, routine fundoplication is not needed when dealing with parahiatal hernia, as the patient does not usually suffer from gastroesophageal reflux. In contrast to parahiatal hernia, hiatal hernia repair is preferred to be done primarily due to the high risk of mesh erosion to the oesophagus, as the mesh is in direct contact with the oesophagus, and various case reports have confirmed this. Stadlhuber et al. published a case series of 28 cases and showed that the complication rate of hioplasty using mesh is higher than previously thought [11]. Although it leads to a lower recurrence rate, complications such as intraluminal mesh erosion and oesophagal stenosis are devastating, and patients have to be treated with total gastrectomy and oesophagectomy.

## Conclusions

True parahiatal hernia is a rare clinical entity that warrants a high index of suspicion. Symptomatic parahiatal hernia in a good surgical candidate warrants surgical repair and is best repaired with mesh placement. The laparoscopic approach to repair is favoured, with promising outcomes. A gastropexy procedure may reduce the recurrence rate and prevent close contact of the mesh with adjacent viscera.
